# Scalable process development of NK and CAR-NK expansion in a closed bioreactor

**DOI:** 10.3389/fimmu.2024.1412378

**Published:** 2024-07-24

**Authors:** Xuening Wang, Maeve Elizabeth Byrne, Chang Liu, Minh Tuyet Ma, Dongfang Liu

**Affiliations:** ^1^ Department of Pathology, Immunology and Laboratory Medicine, New Jersey Medical School, Rutgers-The State University of New Jersey, Newark, NJ, United States; ^2^ Center for Immunity and Inflammation, New Jersey Medical School, Rutgers-The State University of New Jersey, Newark, NJ, United States

**Keywords:** NK cell, CD147-IL15-CAR-NK, expansion, HCC, bioreactor

## Abstract

Production of large amounts of functional NK and CAR-NK cells represents one of the bottlenecks for NK-based immunotherapy. In this study, we developed a large-scale, reliable, and practicable NK and CAR-NK production using G-Rex 100M bioreactors, which depend on a gas-permeable membrane technology. This system holds large volumes of medium with enhanced oxygen delivery, creating conditions conducive to large-scale PBNK and CAR-NK expansions for cancer therapy. Both peripheral blood NK cells (PBNKs) and CAR-NKs expanded in these bioreactors retained similar immunophenotypes and exhibited comparable cytotoxicity towards hepatocellular carcinoma (HCC) cells akin to that of NK and CAR-NK cells expanded in G-Rex 6 well bioreactors. Importantly, cryopreservation minimally affected the cytotoxicity of NK cells expanded using the G-Rex 100M bioreactors, establishing a robust platform for scaled-up NK and CAR-NK cell production. This method is promising for the development of “off-the-shelf” NK cells, supporting the future clinical implementation of NK cell immunotherapy.

## Introduction

Chimeric antigen receptors (CARs) are engineered fusion proteins designed for the specific recognition of a cell surface protein and the initiation of subsequent signaling pathways to kill the specific target cells (1, 2). The recent development of the CAR-T cell is a novel effective strategy to promote anti-tumor therapy. However, CAR-T therapies come with noticeable side effects, including the risk of graft vs host disease (GVHD) and cytokine release syndrome (CRS) (3, 4). The use of CARs has expanded to other cell types, such as NK cells and macrophages (5). NK cells play a critical role in immune activation and surveillance against tumors or abnormal cells (6). Unlike T cells, NK cells show specific cytotoxicity towards target cells without requiring prior sensitization. Importantly, NK cells exhibit minimal or no graft versus host disease (GvHD), which makes them suitable for the development of an “off-the-shelf” therapeutic product (7, 8). NK cells expressing CAR receptors (CAR-NK) have now been recognized as a potent tool against both solid and liquid tumors (9, 10). Various trials have also shown that CAR-NK cells rarely cause CRS like CAR-T cells (11). However, it is challenging to generate enough functional, non-exhaustive NK and CAR-NK for clinical use (7, 12). The development of “off-the-shelf” product involves several key steps, such as the process of freezing and thawing NK cells, which is commonly employed for storage and transportation. However, the freezing and thawing process of NK and CAR-NK cells can result in reduced cell viability and cytotoxicity. Maintaining the functional integrity of NK cells during the freeze-thaw cycle is crucial for their successful application in therapeutic settings.

Developing optimized methods for scaled-up NK expansion is pivotal for advancing the widespread application of CAR-NK in both translational research laboratories and clinical settings. Some feeder expansion systems employed for increasing NK cell numbers and functions face drawbacks, such as low cell expansion rates and the development of an exhaustive phenotype (13, 14). The effectiveness of feeder cells in supporting NK cell growth needs improvement to ensure a consistent and robust expansion. Researchers are actively exploring alternative feeder cell types and optimizing culture conditions to address these limitations, aiming to enhance the efficiency and sustainability of NK cell expansion systems.

Large-scale production of NK cells typically takes at least 14–21 days, and the conventional manual production method involves frequent replenishment of fresh medium and cytokines. This is necessary to maintain an optimal environment for the growth of NK cells, but it unavoidably raises the risk of introducing potential contaminants. The G-Rex-100M closed system, wherein cells remain shielded from the open environment, was specifically crafted to avoid contamination risk (15).

Human peripheral blood mononuclear cells (PBMCs) represent a prominent reservoir of NK cells. However, the fraction of NK cells within PBMCs is modest, typically ranging from 5% to 20% (16). Various strategies have emerged to augment the population of NK cells derived from PBMCs, including the utilization of cytokines, feeder cells, or membrane particles to induce *in vitro* expansion. Notably, these expansion systems exhibit different levels of efficiency in expanding NK cells. One method uses membrane-bound IL-15 and 4–1BB on K562 feeder cells, resulting in significant NK cell expansion. Another method replaces IL-15 with membrane-bound IL-21 on K562 feeder cells (K562-mbIL21), preserving the telomere length of expanded NK cells and leading to a more sustained proliferative potential (17–20). NK cells have recently been expanded using a hyaluronic acid-based biodegradable polymeric scaffold with a macro-porous 3D structure, which supported NK cell proliferation and persistence (21).

Recently, we have successfully developed a new expansion system for NK and CAR-NK cells, utilizing 721.221-mIL21 feeder cells (22). To facilitate the translation of these promising cell therapies from the research laboratory to clinical production, it is crucial to convert validated production approaches into practical methodologies capable of producing large quantities of functional cells.

To address these challenges, we have enhanced both the expansion and transduction process by incorporating the G-Rex-100M closed system, which could produce large-scale high-purity NK cells in a short period. To generate CAR-NK cells, we used CD147 as a valid target for hepatocellular carcinoma (HCC) (10). The expanded CD147-IL15-CAR-NK cells using the closed expansion system effectively killed CD147-positive HCC cell lines *in vitro* and *in vivo* studies. The freeze/thaw process had minimal effect on CD147-IL15-CAR-NK cell cytotoxicity.

This refinement involves optimizing the co-culture ratio of 721.221-mIL21 feeder cells with PBMC cells and cultivating cells within the G-Rex-100M cell growth platform. This strategic implementation not only addresses the hurdles associated with NK cell expansion and CAR-NK cell transduction, but also ensures the generation of clinically relevant cell numbers without exhaustion in a manner compliant with regulatory standards.

## Methods and materials

### Antibodies for flow cytometry

FITC, BV605, PE/Cy7, and BV510 anti-human CD56 antibody (clone HCD56), PE and APC anti-human CD3 antibody (clone OKT3), PE/Cy7 anti-human CD8a antibody (clone HIT8a), BV421 anti-human CD335 (NKp46) antibody (clone 9E2), PE/Cy7 anti-human CD244 (2B4) antibody (clone C1.7), APC anti-human CD366 (Tim-3) antibody (clone F38–2E2), PerCP/Cy5.5 anti-human CD94 (clone DX22), PerCP/Cy5.5 anti-human TIGIT antibody (clone A15153G), FITC anti-human LAG-3 antibody (clone 11C3C65), and were purchased from BioLegend (San Diego, CA, USA). APC anti-human CD16 antibody (clone B73.1), FITC anti-human CD3 antibody (clone UCHT1), BV711 anti-human CD314 (NKG2D) antibody (clone 1D11), PE anti-human HLA-Class I (clone DX17), and FITC anti-human CD107a antibody (clone H4A3) were purchased from BD Biosciences (San Jose, CA, USA). FITC anti-human KIR/CD158 antibody (clone 180704), PE anti-human KIR2DL1 antibody (clone 143211), APC anti-human NKG2A/CD159a antibody (clone 131411), and PE anti-human NKG2C antibody (clone 134591) were purchased from R&D Systems. AF647 Goat anti-human IgG(H+L) F(ab)_2_ fragment antibody was purchased from Jackson ImmunoResearch (West Grove, PA, USA).

### Cell lines and cultures

721.221 wild-type cell line was a gift from Dr. Eric O. Long (National Health of Allergy and Infectious Diseases in National Health of Institutes). 293T, Sk-Hep1, Huh7, and K562 cell lines were purchased from the American Type Culture Collection (ATCC). 721.221-mIL21 cells were made in this lab as previously described (22). 721.221-mIL21 cells were cultured in RPMI-1640 (Corning) supplemented with 10% fetal bovine serum (FBS) and 100 U/mL Penicillin-Streptomycin (Corning) at 37 °C with 5% (v/v) CO_2_. For NK cell expansion, 721.221-mIL21 cells were collected and irradiated at a dose of 10,000 Rad, washed with PBS, and then used as feeder cells for NK cell expansion. 293T was cultured in DMEM (Corning) supplemented with 10% fetal bovine serum (FBS) and 100 U/mL Penicillin-Streptomycin (Corning) at 37°C with 5% (v/v) CO_2_. Cells were frozen in Cell Freezing Medium – DMSO Serum Free (Sigma Aldrich, C6295).

### Primary NK cell expansion

PBMCs were isolated from the buffy coats purchased from the New York Blood Center by using a Lymphocyte Separation Medium (Corning) as described previously (22). For PBNK cell expansion, 5 × 10^6^ PBMCs were cultured with 1 x 10^7^ irradiated feeder cells in 30 ml RPMI-1640 media with 10% Fetal Bovine Serum (Corning), 2mM L-Glutamine (Corning), 100 U/ml Penicillin-Streptomycin (Corning), 200 U/ml IL-2 (PeproTech), and 5 ng/ml IL-15 (PeproTech) in G-Rex 6W cell culture plates (Wilson Wolf 80240M), media was changed every 3-5 days. For G-Rex 100M series bioreactors (Wilson Wolf RU81100 and RU81100-CS), 5–6 × 10^7^ PBMCs were cultured with 1 x 10^8^ irradiated feeder cells in 300 ml RPMI-1640 with 10% FBS along with 200 U/ml IL-2 and 5 ng/ml IL-15. Media were changed every 7–10 days. Total cell numbers were counted using Trypan Blue by an automated cell counter (Nexcelom, MA). To determine the percentage of NK cells, cells were stained for CD3-PE and CD56-APC, followed by flow cytometry analysis. The live and dead cell staining kit (Invitrogen, Cat #L34975A) was used to stain live and dead cells, which then analyzed by flow cytometry.

### Transduction of expanded NK cells with CD19-IL15-CAR and CD147-IL15-CAR

To produce CD19-IL15-CAR or CD147-IL15-CAR retrovirus, 293T cells were transfected with a combination of plasmids containing either CD19-IL15-CAR or CD147-IL15-CAR in SFG backbone, RDF, and PegPam3, as previously described (23). NK cells were harvested on day 4 of expansion and transduced with either CD19-IL15-CAR or CD147-IL15-CAR retrovirus in plates coated with RetroNection (Takara Bio, San Jose, CA). Two days later, cells were transferred to a G-Rex-6W cell culture plate or G-Rex-100M-CS bioreactor and maintained in RPMI-1640 media with 200 U/ml IL-2 (PeproTech) and 5 ng/ml IL-15 (PeproTech). Culture media were changed every 3–4 days and 1 × 10^7^ cells were kept in the wells for expansion. Total cell numbers were counted using Trypan Blue by an automated cell counter (Nexcelom, MA). To determine the percentage of NK cells and expression of CAR, cells were stained for CD3, CD56, and anti-human IgG(H+L) F(ab)_2_ fragment and analyzed by flow cytometry.

### Collecting CAR-NK cells using GatheRex

The transduced PBNK cells were allowed to expand for 14–17 days and then harvested using the GatheRex cell harvest pump (Wilson Wolf). First, G-Rex is pressurized to drain 90% of the medium above cells to concentrate cells, then resuspend the cells with the residual medium in the G-Rex. The pump is then turned on again to drain cells into cell collection bags. The residual cells in the G-Rex were used to measure cell number, access viability, and perform functional assays and *in vivo* animal study.

### Flow cytometry analysis

PBMCs and expanded NK cells were stained with fluorescence-conjugated antibodies in FACS staining buffer (PBS with 1% FBS) on ice for 30 minutes, washed with 1xPBS twice, and samples were analyzed on a FACS LSRII or an LSR Fortessa flow cytometer (BD). PMT voltages were adjusted, and compensation values were calculated before data collection. Data were collected using FACS Diva software (BD) and then analyzed using FlowJo software (BD).

### CD107a degranulation assay

The CD107a degranulation assay was performed as described previously (24). Briefly, expanded NK or CAR-NK cells (5 × 10^4^) were unstimulated, incubated with 1.0 × 10^5^ target cells, Sk-Hep1 or K562 cells in V-bottomed 96-well plates in complete RPMI-1640 media or 100 ng/ml PMA plus 1µg/ml ionomycin as positive control at 37 °C for 2 hours. The cells were harvested, washed, and stained for CD3-PE, CD56-APC, and CD107a-FITC in the presence of 1 µl/ml Golgi-Stop for 30 minutes, and analyzed by LSR Fortessa flow cytometry (BD).

### ^51^Cr release assay

To evaluate the cytotoxic activity of PBNK or CAR-NK cells, the standard 4-h ^51^Cr release assay was used as described before (10). Briefly, target cells SK-Hep1, Huh7, or K562 were labeled with ^51^Cr at 37°C for 2 h and then resuspended at 2 × 10^5^/mL in RPMI1640 medium with 10% FBS. Then, 1 × 10^4^ target cells were incubated with serially diluted PBNK or CAR-NK cells at 37°C for 4 h. After centrifugation, the supernatants were collected and the released ^51^Cr was measured with a gamma counter (Wallac, Turku, Finland). The cytotoxicity (as a percentage) of effector cells was calculated as follows: [(sample − spontaneous release)/(maximum release − spontaneous release)] × 100.

### Xenograft *in vivo* study in NSG mouse model

Mouse xenograft experimental protocols were approved by the Rutgers Institutional Animal Care and Use Committee (IACUC). Animals were maintained and evaluated under pathogen-free conditions following IACUC guidelines. NSG mice from The Jackson Laboratory (Bar Harbor, ME) were used for the *in vivo* experiments. To establish an HCC cell line-derived xenograft (CDX) model, both male and female NSG mice (8-week-old) were injected subcutaneously with 3 × 10^6^ SK-Hep1 cells in 100 μL of PBS mixed with Matrigel Matrix in the right flank. When there was a palpable tumor around day 3, mice were randomly allocated into three groups. Beginning treatment on day 5, cryopreserved CD147-IL15-CAR-NK cells generated from either G-Rex-6W, or G-Rex-100M-CS bioreactor were washed and resuspended in PBS supplemented with IL-2 (10,000 U/mouse) for tail vein intravenous injections with 5 × 10^6^ CD147-IL15-CAR-NK cells in 100 μL PBS. Control groups were infused with PBS buffer. All cells were injected (i.v.) with IL-2 (10,000 U/mouse). The injection was repeated on day 7, day 9, day 16, and day 18. Animal weights were measured and collected twice a week. The tumor size of each NSG mouse was measured by a caliper. Both greatest longitudinal diameter (length) and the greatest transverse diameter (width) were recorded. The tumor size was calculated as follows: tumor size (mm^3^) = ½ (length × width^2^). When the tumor size is more than 2000 mm^3^ or the animal’s weight is reduced by more than 20%, mice were euthanized according to Rutgers IACUC guidelines. The animal survival data were recorded simultaneously.

### Statistical analysis

Data were represented as means ± SEM. The statistical significance was determined using a two-tailed unpaired Student t-test, a two-tailed paired Student t-test, and a one-way ANOVA, where indicated. P < 0.05 was considered statistically significant.

## Results

### Comparison of PBNK cell expansion with PBMC in G-Rex-6W with G-Rex-100M-OS and G-Rex-100-CS bioreactors

**Figure 1:**
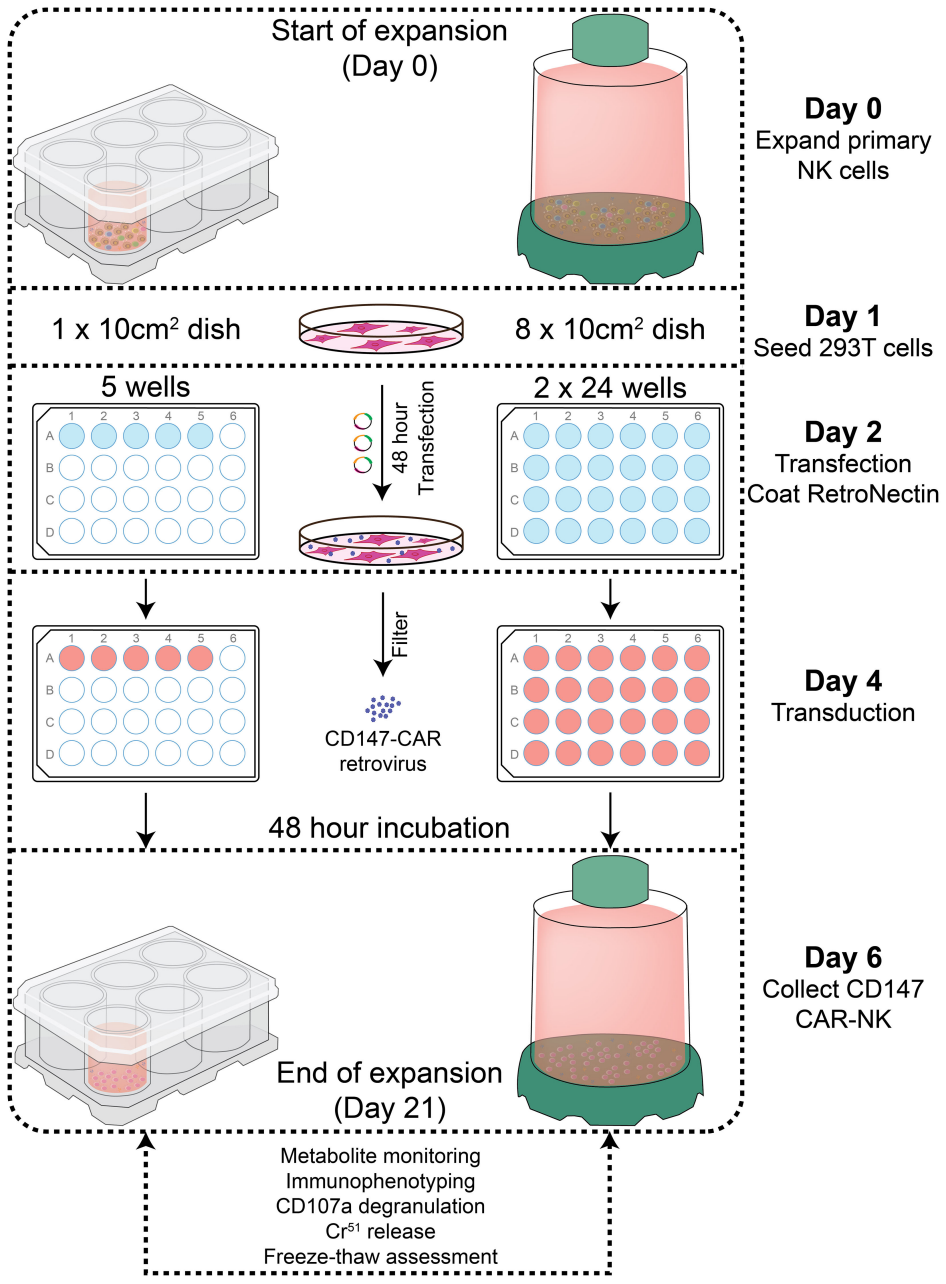
Diagram of PBNK and CD147-IL15-CAR-NK expansion protocol and experimental designs. primary human NK cell expansion with 721.221-mIL21 feeder cells. Feeder cells were irradiated with a dose of 100 Gy (10,000 rad), and then PBMCs were co-cultured with irradiated feeder cells with IL-2 and IL-15 for PBNK cell expansion. In parallel, CD147-IL15-CAR retrovirus was produced by transfecting into 293T cells. The expanded NK cells were transduced with CD147-IL15-CAR retrovirus on day 4 of NK expansion in 24 well plates. Cells were transferred from transduction to the culture system on day 6 and cultured for at least 21 days in either G-Rex-6W or G-Rex-100M bioreactors, then expanded PBNK and CAR-NK cells were subjected to various functional assays.

**Figure 2:**
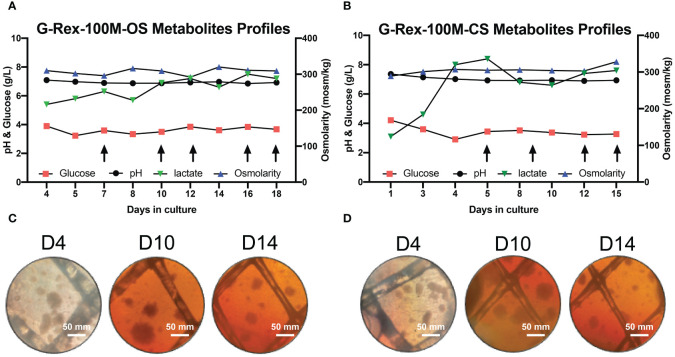
Metabolite’s profiling of PBNK expansion in G-Rex-100M-OS and G-Rex-100M-CS bioreactors. Glucose, pH, lactate, and osmolarity of culture medium in G-Rex-100M-OS **(A)** and G-Rex-100M-CS **(B)** were measured at indicated times to monitor cell proliferation status. Culture Medium was changed (arrows) to maintain metabolite levels for cell expansion. Both lactate and pH levels were kept at stable levels during the whole culture period, glucose levels remain above 3.2 g/L for either PBNK or CAR-NK cells expansion period. The microscope images of cell culture in G-Rex-100M-OS **(C)** and G-Rex-100M-CS **(D)** on day- 4, 10, and 14 post-NK cell expansion images were taken at 10x magnification. scale bars represent 50 mm.

**Figure 3:**
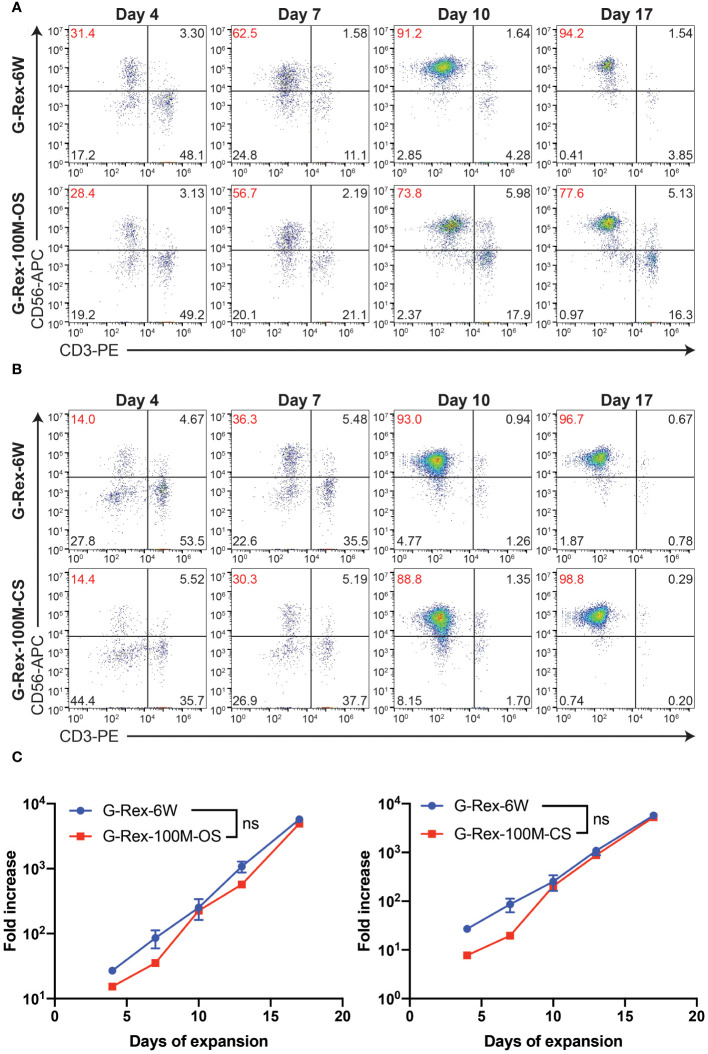
PBNK expansion in G-Rex-100M-OS or G-Rex-100M-CS bioreactors. Representative flow cytometry plots of the purity of PBNK cells expanded with G-Rex-6W plate, G-Rex-100M-OS **(A)**, or G-Rex-100M-CS **(B)** bioreactor at indicated days after cell expansion. PBMCs were stimulated with irradiated 721.221-mIL21 cells on day 0. The purities of PBNK cells were determined at the indicated time points. **(C)** Dynamic time-lapsed expansion data of the fold increase of PBNK cells from different bioreactors with 721.221-mIL21 feeder cells were plotted for the indicated time points.

Our laboratory has already established the procedures for PBNK and CAR-NK expansion using irradiated 721.221-mIL21 feeder cells system in the G-Rex-6W plate (22). To scale up the production of PBNK cells more efficiently, we first started to optimize the process parameters with G-Rex-100M-OS to evaluate the expansion ability of our 721.221-mIL21 feeder system in this bioreactor. To expand human primary NK cells from peripheral blood (PBNK cells), PBMCs were isolated from the buffy coats of healthy donors and co-cultured with irradiated feeder cells along with 200 U/mL IL-2, and 5 ng/mL IL-15. To compare their relative ability to stimulate NK cell expansion, expansions in G-Rex-6W and G-Rex-100M-(OS or CS) were performed in tandem (**Figure 1**). The starting number of PBMCs was 5 million for G-Rex-6W and 50 million to 60 million for the G-Rex-100M bioreactor. Since glucose consumption and lactate secretion are indications of NK cell growth, we monitored the cell proliferation conditions by periodically measuring the medium levels of several metabolic markers, glucose, pH, lactate, and osmolarity (**Figure 2**). To maintain the optimal conditions for NK expansion in the G-Rex-100M-(OS or CS) series device, we started to change the medium when the glucose level fell below 3 g/L, and the lactate level increased above 8 g/L. We observed that lactate levels fluctuated more drastically in the G-Rex-100M-CS system before and after medium changes compared to that of the G-Rex-100M-OS system (**Figure 2B**). Microscopic images of PBNK cell culture for days 4, 10 and 14 in both types of bioreactors were shown (**Figures 2C, D**). NK expansion profiles for the G-Rex-100M-(OS or CS) device gated on CD56 and CD3 by flow cytometry were shown at different time points (days 4, 7, 10, and 17) (**Figure 3A**). A closed system with semi-automated liquid handling in conjunction with the GatheRex Pump could greatly reduce the contamination risk. Next, we tested the expansion in the G-Rex-100-CS (**Figure 3B**). The expansions in both G-Rex-100M-OS and G-Rex 100M-CS bioreactors had similar fold increases of NK cells when compared to the expansion in the G-Rex-6W system (**Figure 3C**). Thus, the NK expansion rates, NK purity, NK metabolic markers among G-Rex-6W plate, G-Rex-100M-OS (open system), and G-Rex-100-CS (closed system) bioreactors are comparable.

### Generation of CD147-IL15-CAR-NK cells in G-Rex-100M-CS device

**Figure 4:**
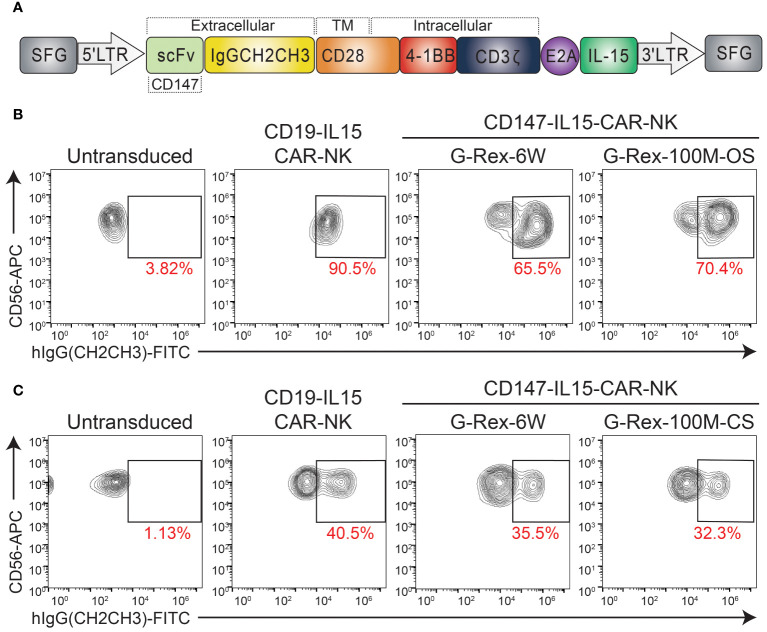
Transduction of CD147-IL15-CAR-NK Cells Using PBNK expanded from G-Rex-100M-OS or G-Rex-100M-CS bioreactors. **(A)** Schematic representation of retroviral vector encoding the CD147-IL15 CAR design. Representative flow cytometry dot plots of the percentage of CD147-IL15-CAR-positive cells. PBMCs were stimulated with irradiated feeder cells and cultured in different devices and transduced with CD147-IL15-CAR retrovirus on day 6 of NK expansion in **(B)** G-Rex-100M-OS or **(C)** G-Rex-100M-CS bioreactor. CD19-IL15-CAR virus was also transduced as a positive control. The transduction efficiency of each CAR vector was shown in the flow charts.

Previous work in this lab has demonstrated the therapeutic potential of CD147-IL15-CAR-modified immune cells for HCC tumors (10). Therefore, we tested if we could generate a scaled-up number of CAR-NK cells in the G-Rex-100M-CS bioreactor. We generated the CD147-IL15-CAR using the SFG retroviral vector (23). The CD147-IL15-CAR contains an anti-CD147 scFv, a human IgG1-CH2CH3 spacer, a transmembrane domain of CD28, the intracellular domain of CD28–4-1BB, the CD3zeta intracellular domain, and the IL15 gene (**Figure 4A**). First, we evaluated this CAR construct using PBNK expanded from both G-Rex- devices. On day 6 of expansion, expanded NK cells were transduced with either CD19-IL15-CAR or CD147-IL15-CAR retrovirus in 24 well plates. CD19-IL15-CAR transduction was used as a control for transduction efficiency. CAR-NK cells were harvested back into the G-Rex-6W or G-Rex-100M-(CS or OS) device 2 days after transduction. CAR-NK cells were cultured in RPMI-1640 with 10% FBS and supplemented with 200U/mL IL-2 and 5 ng/mL IL-15. The percentage of PBNK cells expressing the CD147-IL15-CAR molecules was confirmed by flow cytometry. The transduction efficiency of CD147-IL15-CAR-NK was comparable in NK cells derived from G-Rex-100M-OS device and G-Rex-6W (**Figure 4B**). Next, we repeated the same experiment in the G-Rex-100M-CS device (**Figure 4C**). The transduction efficiency of CD147-IL15-CAR was also comparable in G-Rex-100M-CS devices when compared to the G-Rex-6W plate. In conclusion, both PBNK expansion and CAR-NK production and proliferation could be done efficiently at the scaled-up volume in the G-Rex-100M-CS device as in the G-Rex-6W device.

### Characteristics of expanded PBNK cells and CD147-IL15-CAR-NK cells

**Figure 5:**
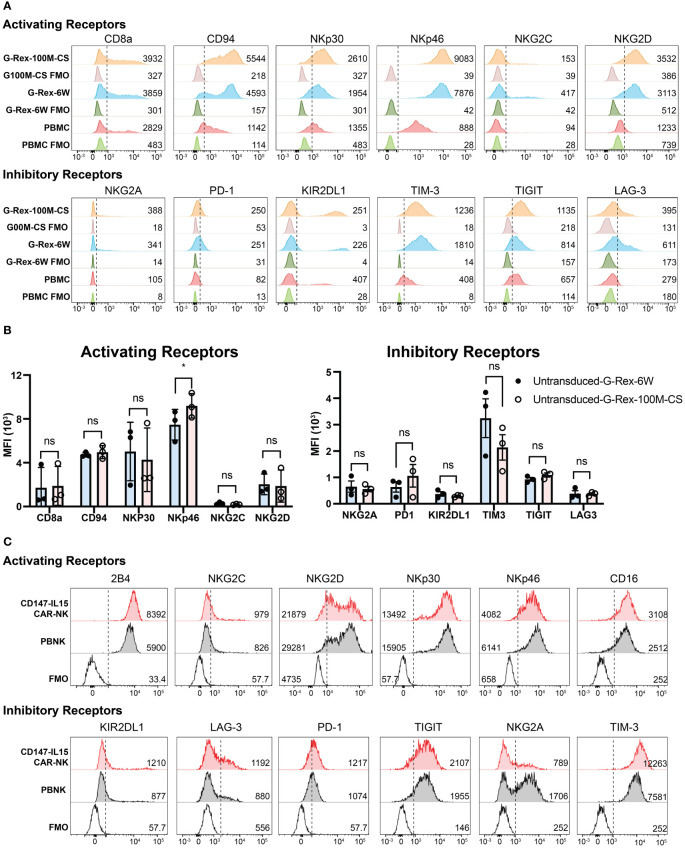
Immunophenotyping of PBNK and CAR-NK expanded with G-Rex-100M-CS bioreactor. **(A)** Representative histograms of the expression of Activating Receptors CD8a, CD94, NKp30, NKp46, NKG2C, NKG2D, and Inhibitory Receptors NKG2A, PD-1, KIR2DL1, Tim3, TIGIT, Lag3 on NK cells expanded from PBMCs using 721.221-mIL-21 feeder cells in either a G-Rex-6W or G-Rex-100M-CS devices. Unexpanded PBMCs were also immunophenotyped as baseline control. Unlabeled PBMC or PBNK cells were used as FMO controls. The mean fluorescence intensity (MFI) is noted in the respective histograms. Respective unstained controls are also represented. **(B)** The summary bar graphs of immunophenotyping of PBNK from three individual blood donors. Data represent the mean ± SEM from three individual donors, * indicates p< 0.01 when compared MFI of surface markers of PBNK cells from different bioreactors; NS, Not significant. **(C)** Representative histograms of the expression of Activating Receptors CD8a, CD94, NKp30, NKp46, NKG2C, NKG2D, and Inhibitory Receptors NKG2A, PD-1, KIR2DL1, Tim3, TIGIT, Lag3 on CD147-IL15-CAR-NK cells transduced from PBNK expanded from G-Rex-100M-CS bioreactor. PBNK cells were immunophenotyped as a control. Unlabeled PBNK cells were used as FMO control. The MFI is noted in the respective histograms.

To further characterize the phenotypes of cell expansions in different bioreactors. We characterized the phenotypes of the cell expansions in different bioreactors by the immunophenotyping of 721.221-mIL21expanded PBNK cells, a flow cytometry panel analysis was used to immunophenotype the cells expanded by the different bioreactors. The activating panel included receptors such as CD8a, CD94, CD16, NKp46, 2B4, NKG2C and NKG2D (**Figure 5A**). The inhibitory panel included receptors such as NKG2A, PD-1, KIR2DL1, TIM-3, TIGIT, and LAG-3 (**Figures 5A**). The expression of these activating and inhibitory receptors on expanded NK cells was comparable between the two bioreactors with slight variations (including higher expression of NKp30 and NKp46). Unstained PBMCs, PBNK-G6W, and PBNK-G100M were used as the baseline control. The changes in the mean fluorescent intensity (MFI) of measured markers are indicated in the figure. The same assays were performed with expanded PBNK cells derived from three biological donors. The MFI of different surface markers resulted in no statistically significant differences between the groups except NKp46 (**Figure 5B**). To further analyze the CD56 subsets in these expanded NK cells, PBMCs were used as a control to establish the different dim/bright populations. The ratios of CD56^dim/bright^ and CD16 expression in G-Rex expanded NK cells determined and compared to those in PBMCs (**Supplementary Figure S1**).

Similarly, the immunophenotypes of CD147-IL15-CAR-NK cells were also analyzed. The expression of activating receptors CD8a, CD94, NKp30, NKp46, NKG2C, NKG2D, and inhibitory receptors NKG2A, PD-1, KIR2DL1, TIM3, TIGIT, LAG3 on CD147-IL15-CAR-NK cells transduced from PBNK expanded from G-Rex-100M-CS bioreactor were shown in different histograms (**Figure 5C**). PBNK cells were used as the control. Interestingly, three activation markers of NK cells, CD16, 2B4, and NKG2C were higher in CD147-IL15-CAR-NK cells compared to PBNK cells, which could be responsible for the higher cytotoxicity effects of CD147-IL15-CAR-NK cells. CD16 and 2B4 are activating receptors on NK cells, higher expression of these markers could correlate to a higher cytotoxicity of the CAR-NK cells as opposed to the naive PBNK cells. Thus, the phenotypes of the NK and CAR-NK cell expansions in different bioreactors are comparable.

### Cytotoxicity of PBNK and CD147-IL15-CAR-NK from G-Rex 100M- (OS or CS) bioreactor

**Figure 6:**
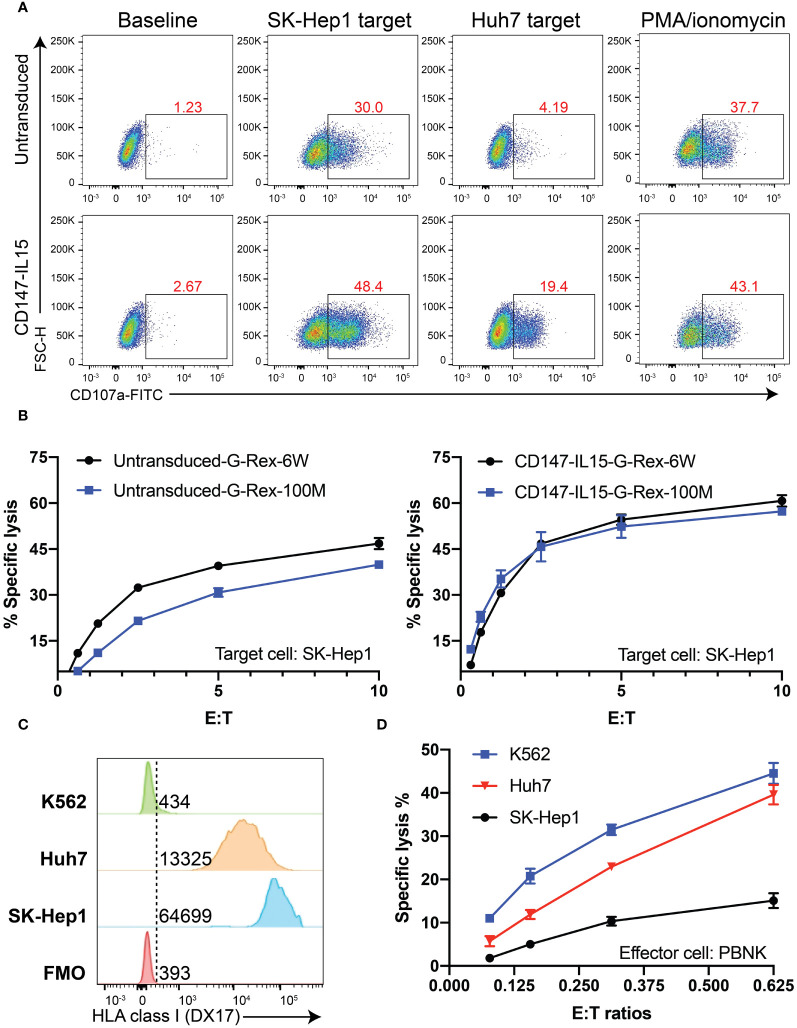
CD147-IL15-CAR-NK cells specifically kill CD147^+^ tumor cells *in vitro*. **(A)** Representative flow cytometric data illustrating CD107a degranulation by PBNK or CD147-IL15-CAR-NK after mixing with the medium (Baseline, negative control), Huh7 cells, SK-Hep1 cells, or PMA/ionomycin (positive control). **(B)** Cytotoxicity of primary PBNK or CD147-IL15-CAR-NK cells derived from different bioreactors using SK-Hep1 target cells was measured by the 4-h standard Cr^51^ release assays. Both CD147-IL15-CAR-NK cells expanded with different G-Rex and showed specific toxicity than PBNK control cells. Data represents the mean ± SEM (n = 3). **(C)** Comparison of HLA Class I expression in SK-Hep1, Huh7, and K562 cells by flow cytometry. **(D)** The comparison of cytotoxicity of PBNK generated from G-Rex-100M-CS towards SK-Hep1, Huh7, and K562 cells. HLA-deficient K562 cells showed better killing than SK-Hep1 and Huh7 cells.

Both CD107a assays and Chromium-51 (^51^Cr) release assays were used to evaluate the activation of CD147-IL15-CAR-NK cells by susceptible target cells. CD107a degranulation assay was used to monitor NK and CAR-NK cell activation. We performed a CD107a degranulation assay by using two HCC cell lines, Huh7 and SK-Hep1, as targets, PBNK showed basal CD107a expression when stimulated with either target cell lines. CD147-IL15-CAR-NK from the G-100M bioreactor showed much higher expression of CD107a when stimulated with HCC cell lines. PMA and Ionomycin stimulation group was used as a positive control (**Figure 6A**). ^51^Cr release assay was used to directly evaluate NK cell killing and whether CD147-IL15-CAR modified immune cells can kill CD147positive HCC cells *in vitro*. To test the cytotoxicity of CD147-IL15-CAR, we used SK-Hep1 target cells for the Cr51 killing assay. CD147-IL15-CAR-NK expanded in both bioreactors had similar killing activities towards Sk-Hep1cells than PBNK cells (**Figure 6B**). To further investigate the role of HLA class I in PBNK-induced killing, we first examined the HLA Class I expression in SK-Hep1, Huh7 and K562 cells (**Figure 6C**). Then Cr^51^ assay was performed with these three tumor cell lines. As expected, HLA-deficient K562 showed better killing compared to other two HLA Class I positive HCC cell lines at lower E:T ratios (**Figure 6D**). Overall, these data show that G-Rex-100M generated CD147-IL15-CAR-NK retains similar cytotoxicity towards HCC cell lines as CAR-NK cells produced from regular G-Rex-6W bioreactor.

### Cryopreserved PBNK cells from the G-Rex-100M-CS bioreactor retain their functions

**Figure 7:**
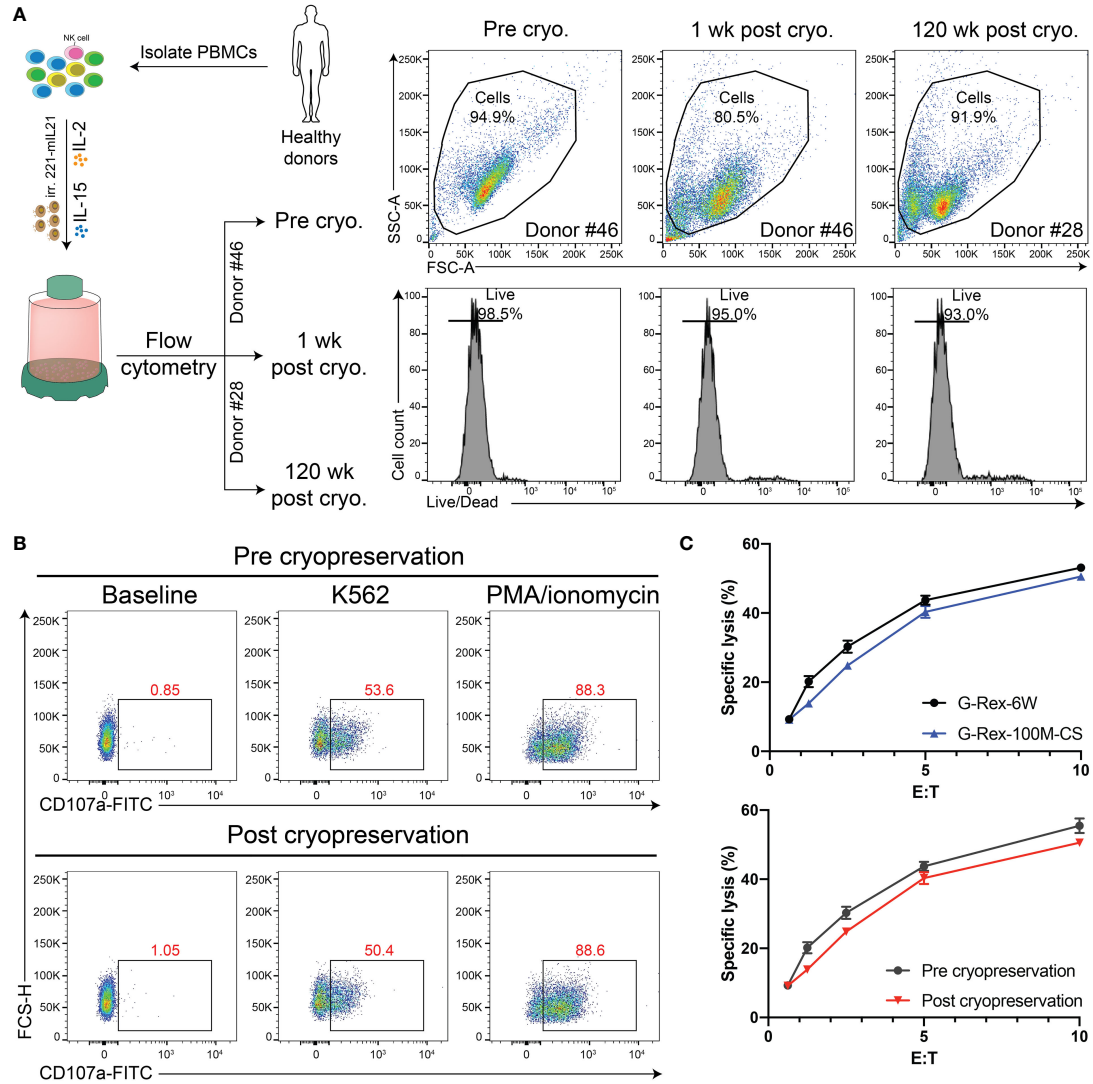
Cryopreservation retains the PBNK purity and function. **(A)** Diagram of experimental design for cryopreservation analysis and comparison. Specifically, PBNK in G-Rex-100M-CS for 14 days, then PBNK cells were frozen in liquid nitrogen for the short term (1-week) or long term (120-week). Cryopreserved cells were thawed, washed in media, and immediately checked for viability with live/dead stains by flow cytometry. **(B)** Representative flow cytometric data illustrating CD107a degranulation by fresh PBNK or cryopreserved PBNK cells. **(C)** The functional activity of these cells was measured by a ^51^Cr release assay. Cryopreserved PBNK cells from G-Rex-100M-CS had similar cytotoxicity as fresh PBNK cells.

It is an essential property that expanded PBNK cells can be frozen and thawed while maintaining a similar phenotype and functions as fresh cells so that these cryopreserved cells can serve as an off-the-shelf cellular product for immediate use. To test whether the scale-up process influences the cell vulnerability to environmental changes, we first evaluated the impact of cryopreservation on the phenotype and viability of thawed cells by flow cytometry. The live/dead fixable dead cell stain kit was used to determine the viability of thawed NK cells (Gating strategy in **Supplementary Figure S2**). Then, we assessed whether the functions of PBNK cells were impacted by performing a Cr^51^ release assay and CD107a degranulation assay. As we expected, with flow cytometry for live/dead staining, we found that both short-term (1 week) and long-term (120 weeks) cryopreservation process had minimal impact on the viability of cells grown in the G-Rex100M-CS bioreactor post-thaw (**Figure 7**). Moreover, freezing of differentiated NK cells *in vitro* did not affect their phenotype, cytotoxicity, and degranulation capacity toward K562 cells (**Figure 7**). We are therefore able to generate large numbers of functional PBNK cells that maintain the same phenotype and function post-cryopreservation, which will allow for their potential use in clinical treatment.

### G-Rex-100M-CS generated CD147-IL15-CAR-NK cells control HCC growth *in vivo*

**Figure 8:**
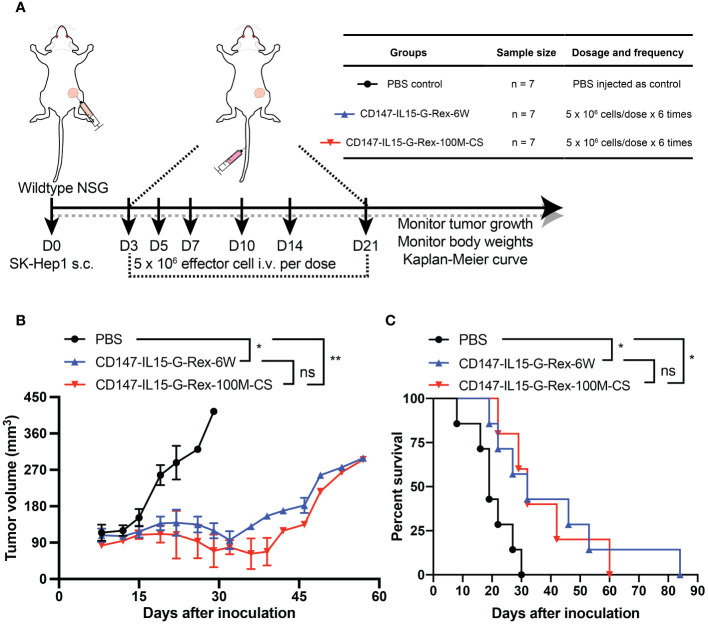
Comparable efficacies of G-Rex-6W and G-Rex-100M-CS generated CD147-IL15-CAR-NK cells. **(A)** Diagram of experimental design of HCC xenograft model. NSG-WT mice were inoculated subcutaneously (SC) with 3 x 10^6^ SK-Hep1 cells for 3 days. 5 x 10^6^ CD147-IL15 CAR-NK cells generated from different bioreactors in addition to 10,000 U of IL-2 per mouse injected through the tail vein. Treatments were administered 6 times on days 3, 5, 7, 10, 14 and 21. Mice were monitored for changes in body weights and tumor sizes. The experimental groups and procedure were summarized in the inserted table. **(B)** Quantitative tumor size of cell line-derived xenograft (CDX) mice treated with CD147-IL15-CAR-NK cells (G-Rex-6W or G-Rex-100M-CS) and PBS (vehicle control group), respectively. All results are mean ± SEM. **(C)** Kaplan–Meier survival curves of tumor-bearing mice after treatment with CD147-IL15-CAR-NK cells groups. The PBS group was used as a negative control. * = p<0.05, ** = p<0.01 when compared between different groups, NS = not significant.

In our previous study, we demonstrated that CD147-CAR can kill HCC in the xenograft NSG model (10). To evaluate whether the G-Rex-100M-CS generated CD147-IL15-CAR-NK cells have similar efficacy, we repeated the experiment in the cell-derived xenograft NSG mice. We first generated CD147-IL15-CAR-NK cells in G-Rex-100M-CS bioreactors till day 14 of cell expansion for cryopreservation. NSG mice were subcutaneously injected with 3 x 10^6^ SK-Hep1 cells premixed with an equal volume of Matrigel on Day 0. Tumor burden was determined by size, and once there was a palpable tumor, 21 mice were randomly assigned to three treatment groups: (1) vehicle control group (PBS), (2) cryopreserved CD147-IL15-CAR-NK (G-Rex-6W; 5 × 10^6^ cells/mouse), and (3) cryopreserved CD147-IL15-CAR-NK (G-Rex-100M-CS; 5 × 10^6^ cells/mouse). On day 3 (D3), mice were tail vein injected with one dose of 5 × 10^6^ cryopreserved CD147-IL15-CAR-NK cells with 10,000 units IL-2, which is important for CAR-NK cell proliferation and cytotoxicity *in vivo*. On days 5, 7, 10, 14, and 21, identical treatments in each group were administered. We compared the efficacies between different G-Rex devices generated by CD147-IL15-CAR-NK cells in the xenograft NSG mouse model. Comparable *in vivo* efficacies measured by median survival between CD147-IL15-CAR-NK (G-Rex-6W) and CD147-IL15-CAR-NK (G-Rex-100M-CS)-injected mice were observed. Mice treated with PBS vehicle control groups developed rapid tumor progression. Mice treated with CD147 CAR NK cells grown in either condition had significantly inhibited tumor progression (P < 0.01), and their median survival was prolonged from 19 days to 32 days. G-Rex-100M bioreactor-generated CAR-NK cells had comparable efficacies (**Figure 8**). The body weights of different treatment groups are comparable (data not shown), which indicates the minimal tolerated toxicity of using CD147-IL15-CAR-NK for the *in vivo* study.

## Discussion

Due to the unique cell biology and immunology features of NK cells, CAR-modified NK cells show less severe side effects than CAR-T cells (7). Both PBNK and CAR-NK cells expanded through the G-Rex 100M-(OS or CS) system show constitutively expressed inhibitory receptors, such as NKG2A, KIR2DL1, and other inhibitory receptors, these features would reduce the off-target effect on normal tissues.

In this study, we also monitored PBNK expansion through the measurement of different key metabolic markers. The increase of lactate levels and pH and the reduction of glucose levels were tightly monitored and used as a guideline for indication of medium exchange. CD147-IL15-CAR-NK cells could also proliferate effectively in the G-Rex 100M-(OS or CS) system. In this manner, we can produce enough highly transduced CAR-NK cells in a short period for the clinical study. Lactate production can increase with higher cell densities due to increased metabolic activities. That is the reason that lactate levels fluctuated before and after the medium exchange in the G-Rex-100M bioreactors.

Our current study utilized fetal bovine serum (FBS) supplemented medium for PBNK and CAR-NK expansion to optimize the expansion conditions with G-Rex-100M bioreactor. However, FBS is not desirable in clinical-grade cell therapy production due to potential immunogenicity concerns (25). Our future studies will investigate the expansion process using FBS-free medium to mitigate the interference of serum-derived components.

This study demonstrated that G-Rex-100M expanded NK and CAR-NK cells can be cryopreserved and retain their cytotoxicity *in vitro* and *in vivo* (**Figure 7**), which is the key to the success of “off-the-shelf”. These results suggested cryopreserved cells can recover quickly and still retain functional to kill tumor cells, such as HCC cells (**Figure 8**). Taken together, even though there is a small portion of cell loss after cryopreservation, the retained portion of cells post-thaw remained cytotoxic towards tumor cells, which was comparable to that of fresh cells. Furthermore, our *in vivo* studies demonstrated that CD147-IL15-CAR-NK generated from different bioreactors had similar efficacy in reducing HCC tumor progression. The median survival for CD147-IL15-CAR-NK treated groups was prolonged from 19 days to 32 days.

In summary, first, this study has demonstrated that G-Rex-100M could expand PBNK and CAR-NK cells at scalable levels efficiently. Second, these expanded cells can be cryopreserved and recovered maintaining potency and antitumor effects *in vitro*. In-depth characterization and comparison to matched fresh samples of this cryopreserved NK cell product established its feasibility for clinical use. This report in combination with recent studies that showed promising results in the cryopreservation of *ex vivo* expanded NK cells that maintain their function post-thaw lays the groundwork for developing a clinical-grade NK-cell product with potentially broad clinical applications. Further studies with *in vivo* animal models also completed the evaluation of the G-Rex-100M system for their application. Therefore, we developed a promising platform for the development of “off-the-shelf” NK and CAR-NK cells, supporting the future clinical implementation of NK and CAR-NK cell immunotherapy in a closed bioreactor.

## Data availability statement

The data supporting the findings of this study are available within the article/**Supplementary Materials**. Requests to access the datasets should be directed to DL, dongfang.liu@rutgers.edu.

## Ethics statement

Ethical approval was not required for the studies on humans in accordance with the local legislation and institutional requirements because only commercially available established cell lines were used. The animal study was approved by Rutgers Institutional Animal Care and Use Committee. The study was conducted in accordance with the local legislation and institutional requirements.

## Author contributions

XW: Data curation, Formal analysis, Investigation, Methodology, Project administration, Validation, Writing – original draft, Writing – review & editing. MB: Data curation, Formal analysis, Methodology, Writing – review & editing. CL: Data curation, Formal analysis, Writing – review & editing. MM: Data curation, Formal analysis, Investigation, Methodology, Validation, Writing – review & editing. DL: Data curation, Formal analysis, Funding acquisition, Investigation, Methodology, Project administration, Supervision, Writing – original draft, Writing – review & editing.
