# Supplementary material for: Scalable process development of NK and CAR-NK expansion in a closed bioreactor
Source: Front Immunol. 2024 Jul 24;15:1412378. doi: 10.3389/fimmu.2024.1412378 (PMC11303211; doi:10.3389/fimmu.2024.1412378)
Supplement: Supplementary file 1 [file DataSheet_1.docx]

**Supplementary data:**

**Scalable Process Development of NK and CAR-NK Expansion in a Closed Bioreactor**

Xuening Wang, Maeve Elizabeth Byrne, Chang Liu, Minh Tuyet Ma and Dongfang Liu


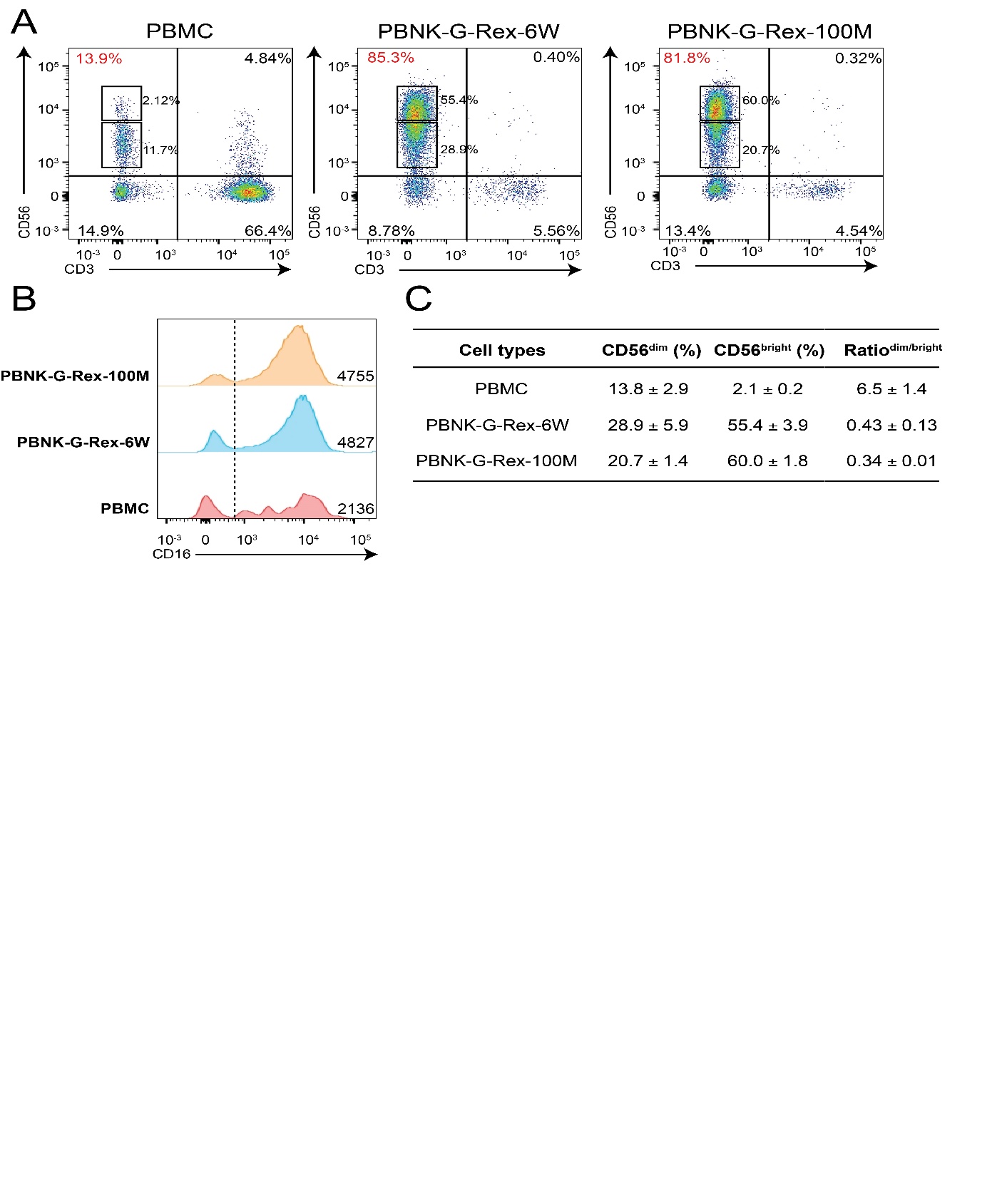


**Supplementary Figure S1: The expression of CD56^+^/CD16^+^ on NK cells expanded in G-Rex bioreactor.** (**A)** Representative dot plots of percentages of CD56^dim/bright^ NK cell populations. PBMC cells were used as a control to set up different dim/bright populations. (**B**) Quantitative histogram data of mean fluorescence intensity (MFI) of CD16 positive NK cells are shown. (**C**) The summary table of CD56dim/bright ratios in PBNK cells derived from different G-Rex bioreactors.


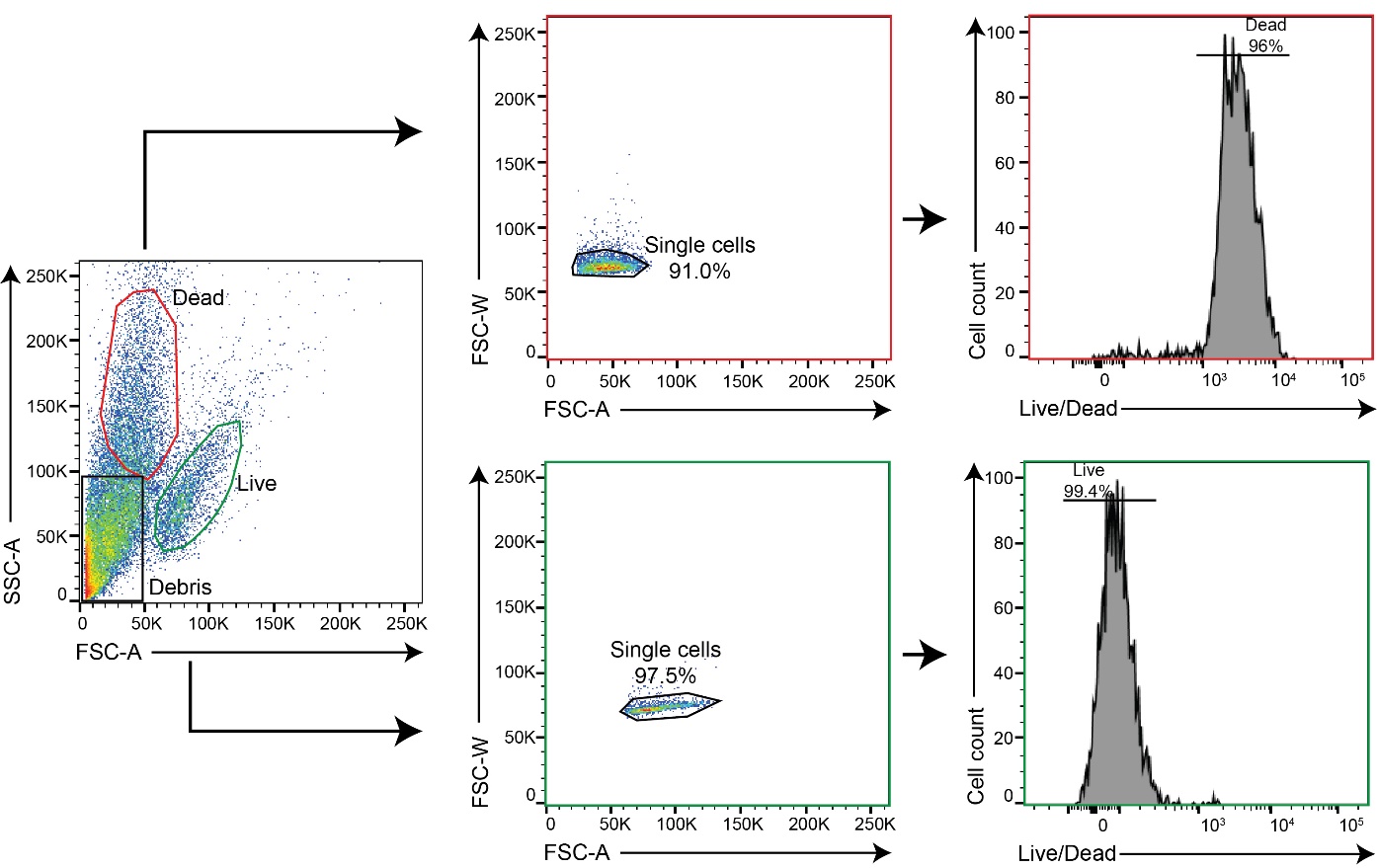


**Supplementary Figure S2: Gating strategy for live/dead cells flow cytometry**. Expanded primary NK cells were cultured without supplemented IL-2 and IL-15 cytokines for 72 hours as a positive live/dead control. Cells were collected and stained with live/dead stain (Invitrogen, Cat #L34975A) at 1:3000 concentration in PBS buffer for 30 minutes on ice. Cells were washed with PBS and resuspended for flow cytometry. Cells were gated on live and dead populations separately as shown to validate the staining. A higher debris and unhealthy cell populations are observed as cells were rested without cytokines in the presence of irradiated 721.221-mIL21 feeder cells.
